# Amnion cell-mediated immune modulation following bleomycin challenge: controlling the regulatory T cell response

**DOI:** 10.1186/scrt542

**Published:** 2015-01-29

**Authors:** Jean L Tan, Siow T Chan, Camden Y Lo, James A Deane, Courtney A McDonald, Claude CA Bernard, Euan M Wallace, Rebecca Lim

**Affiliations:** The Ritchie Centre, Monash Institute of Medical Research, Monash University, 27-31 Wright St, Clayton, Victoria 3168 Australia; Monash Micro Imaging, Monash University, 27-31 Wright St, Clayton, Victoria 3168 Australia; Department of Obstetrics and Gynecology, Monash University, 246 Clayton Rd, Clayton, Victoria 3168 Australia; Australian Regenerative Medicine Institute, Building 75, Wellington Rd, Clayton, Victoria 3168 Australia

## Abstract

**Introduction:**

The immunomodulatory properties of human amnion epithelial cells (hAECs) have been previously described in several disease models. We previously reported on the ability of hAECs to influence macrophage phenotype and chemotaxis. In this study, we aim to elucidate the contribution of regulatory T cells (Tregs) to macrophage polarisation and downstream effects on inflammation and fibrosis in a bleomycin model of lung injury.

**Methods:**

Either CD45^+^/FoxP3^+^*Tregs* or CD45^+^/FoxP3^*-*^*non-Tregs* were adoptively transferred into *Rag1*^*-/-*^ mice immediately prior to bleomycin challenge. Four million hAECs were administered 24 hours later. Outcomes were measured 7 or 14 days later.

**Results:**

Mitigation of lung inflammation and fibrosis was observed only in animals that received both hAECs and Tregs. hAEC treatment also induced the maturation of non-Tregs into FoxP3-expressing Tregs. This event was found to be transforming growth factor-beta (TGFβ)-dependent. Furthermore, polarisation of macrophages from M1 to M2 occurred only in animals that received hAECs and Tregs.

**Conclusions:**

This study provides the first evidence that Tregs are required for hAEC-mediated macrophage polarisation and consequential mitigation of bleomycin-induced lung injury. Uncovering the interactions between hAECs, macrophages, and T-cell subsets is central to understanding the mechanisms by which hAECs elicit lung repair.

**Electronic supplementary material:**

The online version of this article (doi:10.1186/scrt542) contains supplementary material, which is available to authorized users.

## Introduction

Gestational tissues, including the placenta and fetal membranes, are abundant sources of stem cells and stem-like cells. Possibly reflective of the maternal status, these cells also bear potent immunomodulatory properties. Fetal derived mesenchymal stromal cells (MSCs) have a greater ability to suppress antigen-specific T-cell proliferation compared with maternal MSCs [1]. Both amniotic membrane-derived MSCs and human amnion epithelial cells (hAECs) are able to inhibit dendritic cell differentiation [2] We, and others, have previously reported that hAECs exert protective and pro-reparative effects in the settings of lung [3–7], liver [8, 9], and neurological [10–12] diseases. Unlike many stem cells and stem-like cells, hAECs can be isolated from amniotic membranes in numbers sufficient for clinical use (approximately 150 to 200 million) within 6 hours without the need for serial passaging [13, 14]. This may be an advantageous attribute given the recent reports that serial passaging can result in epigenetic changes [15–17] and genomic mutations [18] as well as compromise immunomodulatory capabilities [2].

Although there have been some reports of hAEC engraftment [4, 9], this may not be a major mechanism of hAEC action. Similar to recent reports in the field of MSC research, hAECs appear to exert their effects primarily via paracrine signaling rather than functional cell engraftment. There are now reports describing the biological effects of hAEC-conditioned media, such as the ability to influence the phagocytic ability and polarity of macrophages [10] and the fibrogenic/fibrolytic balance in hepatic stellate cells [19]. Indeed, it appears that hAECs are only able to exert their protective/reparative effects in the presence of functional macrophages [20]. This is perhaps not surprising since endogenous lung macrophages play a central role in the regulation of the immune response to injury. For example, macrophages are able to induce the generation of regulatory T cells (Tregs) from naïve CD4^+^ T cells [21]. Reciprocally, Tregs have been reported to induce a phenotypic and functional switch in macrophage polarity [22].

Given that Tregs have also been shown to be important in resolving lung inflammation and fibrosis by reducing fibrocyte recruitment, we set out to explore whether hAEC treatment altered the Treg population and whether hAEC polarisation of macrophages is dependent on Treg activity. Furthermore, we asked which cytokines were key to the reparative events that we had previously observed. Using transgenic *FoxP3-GFP* mice and *Rag1*^*-/-*^ mice, we performed a series of adoptive transfer studies and *in vitro* co-culture studies to answer these questions. In the recent National Institutes of Health (NIH) workshop on Cell Therapy for Lung Disease, understanding the mechanism of action and identifying cellular targets were listed as research priorities [23]. Furthermore, guidelines from the Therapeutic Goods Administration (Australia) on biologicals, including cell therapies, state that “even where the mechanism of action is not understood in detail, the main effects of the biological should be known” [24]. With these considerations, further delineating immunological events following hAEC administration represents an important step in translating this research to the clinic.

## Methods

### Human amnion epithelial cell isolation

Placentae were collected from consented patients undergoing elective caesarean sections at term. Isolation of hAECs was performed as previously described [14] in accordance with guidelines and approval from the Southern Health Human Research Ethics Committee. Amnions collected had a mean gestational age of 38 weeks. In total, 14 amnions were used in this study: six for animal injections and eight for *in vitro* experiments.

### Mice

**Figure 1:**
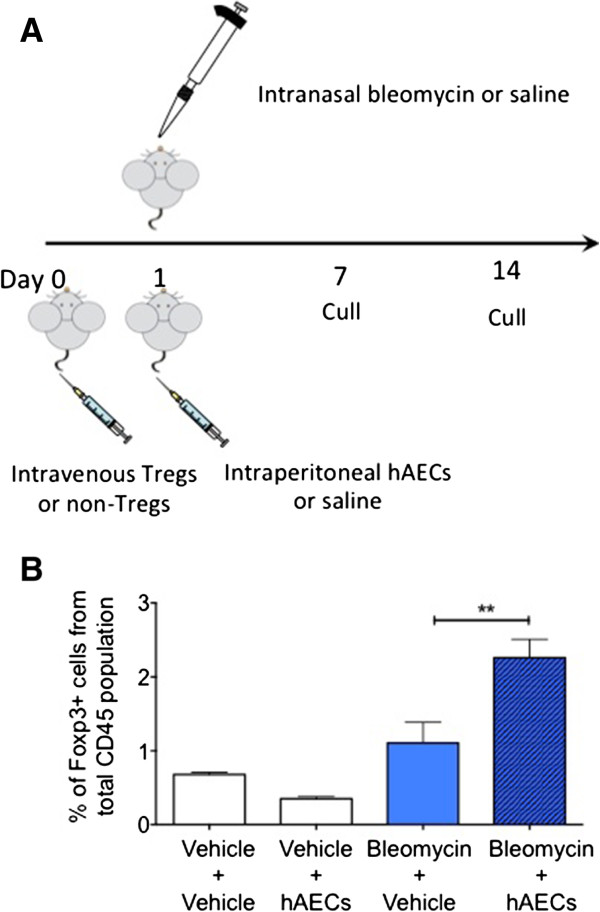
**Human amnion epithelial cell (hAEC)-mediated repair following bleomycin challenge is associated with regulatory T cell (Treg) expansion. (A)** Experimental timeline for basic bleomycin studies. **(B)** The FoxP3+ Treg population was unchanged by bleomycin challenge but was increased in animals that received hAECs. **P <0.01.

All animal experiments were approved by the Monash University Animal Ethics Committee and conducted in accordance with the Australian Code of Practice for the Care and Use of Animals for Scientific Purposes. In total, 150 female mice 8 to 12 weeks old were used. *Rag1*^*-/-*^ mice were sourced from Animal Resources Centre, Australia, and *Foxp3-GFP* mice from Alex Rudensky, University of Washington. Mice were randomly divided into eight groups as listed below and summarised in Figure 1A. On average, six mice were used per group for flow cytometry, seven per group for histology, and six per group for protein and gene analyses. Animals were humanely culled 7 or 14 days later.

**Saline controls:** Intranasal (IN) saline instillation and intraperitoneal (IP) injection of saline 24 hours later;

**Bleomycin controls:** IN bleomycin instillation and IP injection of saline 24 hours later;

**Saline + hAECs:** IN saline instillation and IP injection of 4 million hAECs 24 hours later;

**Bleomycin + hAECs (hAECs alone):** IN bleomycin instillation and IP injection of 4 million hAECs 24 hours later;

**Tregs + bleomycin + saline (Tregs alone):** Intravenous (IV) injection of 0.5 million FoxP3^+^ Tregs and an IN instillation of bleomycin followed by an IP injection of saline 24 hours later;

**Tregs + bleomycin + hAECs (Tregs + hAECs):** IV injection of 0.5 million FoxP3^+^ Tregs and an IN instillation of bleomycin followed by an IP injection of 4 million hAECs 24 hours later;

**non-Tregs + bleomycin + saline (non-Tregs alone):** IV injection of 0.5 million CD45^+^FoxP3^-^ non-Tregs and an IN bleomycin instillation followed by an IP injection of saline 24 hours later;

**non-Tregs + bleomycin + hAECs (non-Tregs + hAECs):** IV injection of 0.5 million CD45^+^FoxP3^-^ non-Tregs and an IN bleomycin instillation followed by an IP injection of 4 million hAECs 24 hours later.

### Isolation of cells for adoptive transfer

Spleens from *Foxp3-GFP* transgenic mice were homogenised to obtain a single cell suspension. Cells were re-suspended in magnetic-activated cell sorting (MACS) buffer after red blood cell lysis. CD4^+^ cells were enriched the CD4^+^ T-cell enrichment kit II (130-095-248; MACS Miltenyi Biotec GmbH, Bergisch Gladbach, Germany) in accordance with the guidelines of the manufacturer. FoxP3^+^ cells (Tregs) were obtained from the CD4^+^ enriched population, whereas CD45^+^ FoxP3^-^ cells were obtained from the CD4^-^ fraction. FoxP3^+^ cells were flow-sorted according to their GFP expression. Gating strategy is described in the online data supplement (Figure S1 in Additional file 1). CD45^+^ (1:300, 560501; BD Biosciences, Franklin Lakes, NJ, USA) were obtained by a flow-sorting Mo-Flow™ XDP High-Speed cell sorter (Beckman Coulter, Brea, CA, USA). Sorted cells were resuspended in saline to obtain 0.5 × 10^6^ cells in 200 μL.

### Tissue collection

After mice were culled, bronchoalveolar lavage fluid was collected from each animal in 1.5 mL of saline by using a 19-gauge blunt needle. Lung tissues were divided equally for measuring immunohistochemical and protein outcomes as previously described [3]. Both lungs were collected from an identical cohort of animals to determine macrophage polarity and function.

### Histological and immunohistochemical analysis

#### Tissue collection and processing

To perfuse the left lung, the right lung was ligated at the mainstream bronchus and 4% paraformaldehyde was instilled into the left lung through an incision in the trachea. The lungs were filled at 20 cm of water pressure. For paraffin-embedded blocks, lung tissue was transferred into 70% ethanol for 24 hours prior to processing. For frozen lung sections, lung lobes were transferred into 30% sucrose solution and stored at 4°C for 48 hours to allow complete diffusion of the solution. The tissues were transferred into 80% Tissue-Tek optimal cutting temperature (OCT) compound (Sakura Finetek, Leiden, Holland) diluted with 30% sucrose solution and kept under vacuum for 24 hours. Tissues were embedded in OCT compound for cryosectioning.

#### Lung morphology

Paraffin-embedded lung sections of 5-μm thickness were stained by using hematoxylin and eosin, and 20 to 25 sequential fields of view (200× magnification) were captured by using brightfield light microscopy (AxioSkop, Carl Zeiss, Oberkochen, Germany) for tissue-to-airspace analysis using the Image J software package (NIH, Bethesda, MD, USA) as previously described [8].

#### F480 immunohistochemistry

Immunohistochemical detection of macrophages was performed as previously described [10]. Immunostaining was performed by using a rat anti-mouse F480 monoclonal primary antibody (1:200, MCA497R; Serotec, Oxford, UK) and biotinylated goat anti-rat IgG (1:100, AP183β; Millipore, Billerica, MA, USA). For analysis, 20 to 25 sequential fields of view (200× magnification) were captured and Metamorph software (Molecular Devices, Sunnyvale, CA, USA) was used to determine total numbers of nuclei and F4/80^+^ cells.

#### Foxp3 immunofluorescence

Immunohistochemical detection of GFP-tagged Foxp3^+^ Tregs was performed by using fluorescent microscopy on cryosections. True staining was confirmed by co-localisation of chicken anti-GFP (1:1,000, GFP-1020; Aves Labs, Inc., Tigard, OR, USA) with GFP over nuclear staining (4′,6-diamidino-2-phenylindole, or DAPI) and absence of auto-fluorescence in the red channel.

#### Lung collagen quantification

Collagen content was determined by using the Sircol™ Collagen Assay (catalogue #S1000; Biocolor, Carrickfergus, UK). Homogenised lung tissues were analysed in accordance with the guidelines of the manufacturer.

#### Flow cytometric analysis

Lung tissues were minced and digested in Waymouths media (Invitrogen, Carlsbad, CA, USA) containing 25 mg/mL collagenase A (Roche Diagnostics, Mannheim, Germany), 2.5 mg/mL of DNase I (Sigma-Aldrich, St. Louis, MO, USA), 25 mM Hepes (Sigma-Aldrich), and 10% heat-inactivated fetal bovine serum (Invitrogen) for 15 minutes at 37°C and 5% CO_2_. Cells were incubated with an Fc-receptor blocker (553141; BD Pharmingen) for 15 minutes on ice and stained with the following antibodies: CD45-V450 (1:150, 560541; BD Biosciences, North Ryde, NSW, Australia), F4/80-PE (1:100, 12-4321-82; eBioscience, San Diego, CA, USA), CD86-PE Cy-7 (1:200, 560501; BD Biosciences, San Jose, CA, USA), and CD206-Alexa Fluor 647 (1:200, 12310; Australian Biosearch, Karrinyup, WA, Australia). Data collected were analysed by using FlowJo cytometric analysis software (Tree Star, Ashland, OR, USA). For gating strategy, see Figure S2 in Additional file 1 in the online data supplement.

### *In vitro*studies

#### CD4^+^ T-cell co-culture with human amnion epithelial cells

Splenocytes from *FoxP3-GFP* mice were obtained as described above. Cells were plated at 2.5 × 10^6^ in the anti-CD3 (MAB484; R&D Systems, Minneapolis, MN, USA, 10 μg/mL) pre-coated bottom chamber of a sixwell plate and treated with anti-CD28 (553294; BD Pharmingen, 2 μg/mL) to activate T cells as previously described [13]. Five million hAECs were added to the top chamber and cultured for 5 days prior to flow cytometry.

#### CD4^+^ T-cell co-culture with human amnion epithelial cell-pretreated macrophages

CD4^+^ T cells were indirectly co-cultured hAEC-pretreated or non-pretreated macrophages for 5 days by using transwell inserts (0.4 μm; BD Falcon, Franklin Lakes, NJ). FoxP3 transcription was determined by flow cytometric detection of GFP expression. Co-culture details are described in the online supplementary methods. Where appropriate, recombinant transforming growth factor-beta (TGFβ) (240-B-001MG/CF; R&D Systems, 5 ng/mL) was added to CD4^+^ FoxP3^-^ (non-Treg) cell cultures as a positive control. To assess the contribution of hAEC-derived TGFβ to Treg expansion, anti-TGFβ blocking antibody (2939, 1 mg/mL; Tocris Bioscience, Bristol, UK) was added to cultures to block TGF-β-mediated Foxp3 expansion.

#### Cytokine analysis

Concentrations of TGFβ1, interleukin-4 (IL-4), and prostaglandin E2 (PGE2) in the culture supernatant were determined by using commercially available kits. TGFβ1 (560429; BD Biosciences, San Diego, CA, USA), IL-4 (MBS705670; MyBiosource, San Diego, CA, USA), and PGE2 (514010; Caymen Chemical, Ann Arbor, MI, USA). Primer sequences used for quantitative polymerase chain reaction are as follows: *IL-4*: forward TGT CCA CGG ACA CAA GTG CGA, reverse TCT CAT GAT CGT CTT TAG CCT TTC C; *Cox 2*: forward CCA CCC GCA GTA CAG AAA GT, reverse CAG GAT ACA GCT CCA CAG CA; and *TGF-β*: forward CCC TGG ACA CCA ACT ATT GC, reverse GCA GAA GTT GGC ATG GTA GC. Cycling conditions were 95°C for 5 minutes, 60°C for 25 seconds for 35 cycles.

#### Statistical analysis

Data were expressed for each experimental group as mean ± standard error of the mean. Statistical significance was determined with GraphPad Prism (GraphPad Software, Inc., San Diego, CA, USA). Differences between two experimental groups were determined by using an unpaired, one-tailed *t* test. Differences across three or more experimental groups were determined by using one-way analysis of variance with the Bonferroni *post hoc* test. Confidence intervals of 95% were deemed significant.

## Results

### Human amnion epithelial cell treatment increased regulatory T cell numbers in the lung

Given the established role of Tregs in the resolution of lung fibrosis [15, 17], we evaluated the effects of hAECs in bleomycin-injured *FoxP3-GFP* mice, where FoxP3^+^ Tregs express GFP. When hAECs were administered 24 hours following a single bleomycin challenge, we observed a significant increase in the percentage of CD45^+^ FoxP3^+^ cells (Tregs) in the lung 7 days following bleomycin challenge (Figure 1B, 2.26 ± 0.25 versus 1.11 ± 0.28, *P* = 0.006). We suggest that this observation is consistent with the association of hAEC-mediated lung repair with either the recruitment of peripheral Tregs to the lung or the inducement of the *in vivo* expansion or conversion of resident Tregs within the lung.

### Human amnion epithelial cells require regulatory T cells to mitigate bleomycin-induced lung fibrosis in Rag1^-/-^ mice

**Figure 2:**
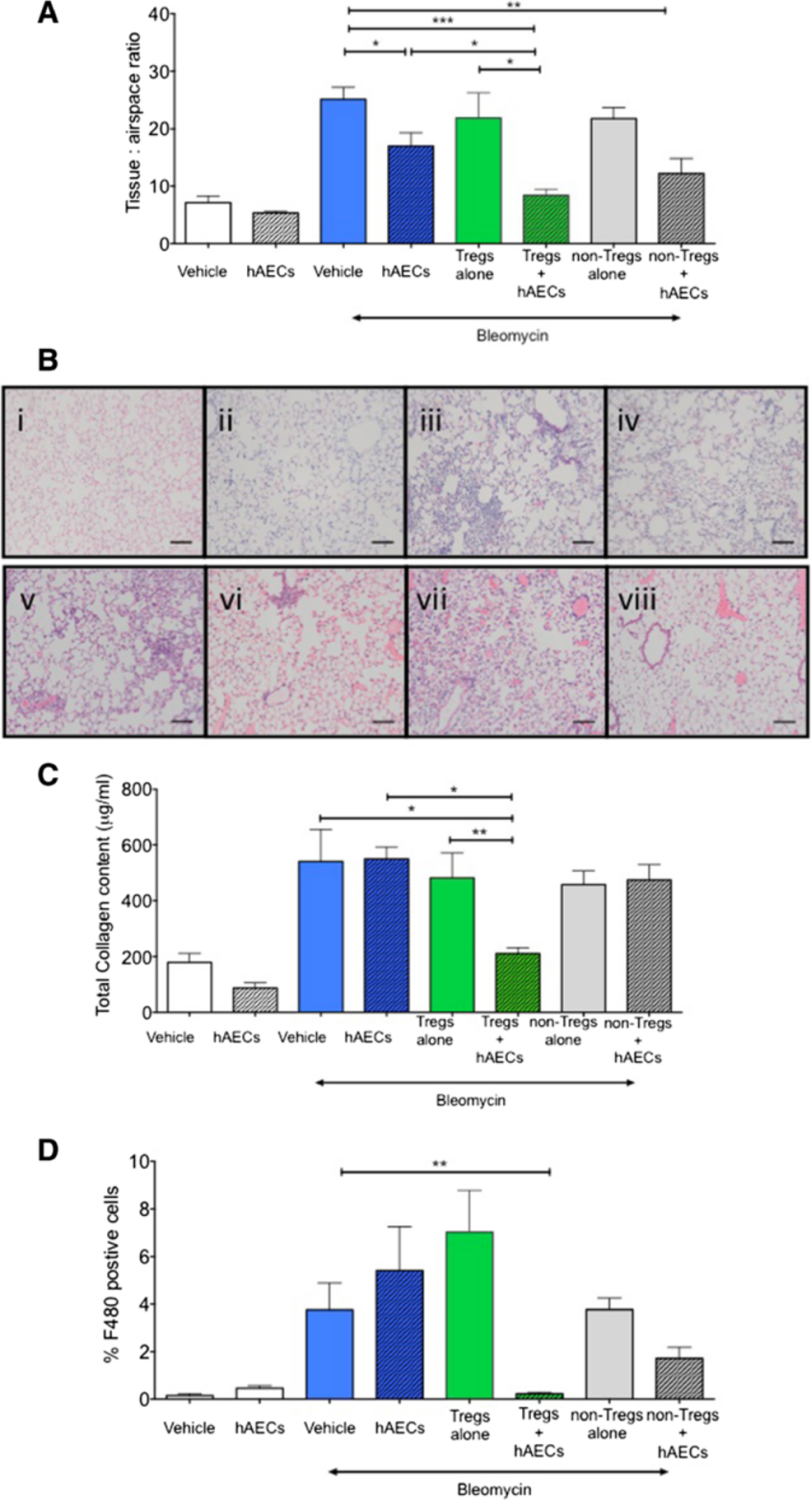
**Regulatory T cells (Tregs) are required for human amnion epithelial cell (hAEC)-mediated repair of bleomycin-induced lung injury in Rag1** ^**-/-**^ **mice. (A)** Administration of bleomycin into *Rag1* ^*-/-*^ mice resulted in a significant increase in tissue-to-airspace ratio. A small reduction was observed following hAEC administration alone. Tissue-to-airspace ratio was lowest in the Treg + hAEC group, and this was significantly lower compared with groups given either Tregs or hAECs (*P <0.05, **P <0.01, ***P <0.001). **(B)** Representative images of hematoxylin-and-eosin-stained lung sections were taken at 200× magnification (scale bar = 100 μm). (i) Saline, (ii) saline hAECs, (iii) bleomycin, (iv) bleomycin hAECs, (v) CD45^+^ bleomycin saline, (vi) CD45^+^ bleomycin hAECs, (vii) Tregs bleomycin saline, (viii) Tregs bleomycin hAECs. **(C)** At day 14, bleomycin administration significantly increased total lung collagen content. Co-administration of Tregs and hAECs significantly reduced lung collagen content in comparison with control groups given hAECs or Tregs alone (*P <0.05, **P <0.0001). **(D)** Macrophages were identified by F4/80 staining and were quantified at day 7. Bleomycin administration resulted in a significant increase in macrophage numbers, but administration of hAECs alone had no significant effect. The number of macrophages in the lungs was reduced only in the Treg + hAEC group (**P <0.01).

To examine the role of Tregs in hAEC-mediated lung repair, we administered hAECs into bleomycin-challenged *Rag 1*^*-/-*^ mice and assessed changes to the tissue-to-airspace ratio, an indicator of lung architectural damage and inflammatory cellular infiltrate. Fourteen days following bleomycin challenge, tissue-to-airspace ratio and collagen content in the lungs increased as expected. hAEC treatment modestly reduced the tissue-to-airspace ratio in *Rag 1*^*-/-*^ mice (Figure 2A, 24.06 ± 2.23 versus 17.00 ± 2.35, *P* = 0.017). Representative images of lung histology are depicted in Figure 2B. Collagen content remained unchanged by hAEC administration (Figure 2C, 550.3 ± 41.82 versus 540.9 ± 115 μg/mL, *P* = 0.94).

When Tregs were adoptively transferred prior to hAEC administration (Treg + hAEC), both tissue-to-airspace ratio and lung collagen content were markedly reduced compared with bleomycin controls (tissue-to-airspace ratio 8.4 ± 1.0 versus 27.26 ± 2.7, *P* = 0.002; collagen content 210.0 ± 10.9 versus 540.9 ± 115.0 μg/mL, *P* = 0.02). Tissue-to-airspace ratio and lung collagen content were also lowered in the Treg + hAEC group compared with hAECs alone (tissue-to-airspace ratio 8.4 ± 1.0 versus 17.0 ± 2.4, *P* = 0.01; collagen content 210.0 ± 10.9 versus 550.3 ± 41.9.0 μg/mL, *P* <0.0001), suggesting that Tregs play a role in hAEC-mediated mitigation of bleomycin-induced lung injury. These effects were not observed in animals receiving only adoptively transferred Tregs and no hAECs (tissue-to-airspace ratio 21.88 ± 5.42, *P* = 0.31; collagen content 481.2 ± 89.3, *P* = 0.69). The adoptive transfer of CD45^+^FoxP3^-^ cells (hereafter referred to as ‘non-Tregs’) prior to hAEC treatment was also associated with some mitigation of increased tissue-to-airspace ratio (12.23 ± 2.61, *P* = 0.002) but not collagen deposition (473.9 ± 55.63 μg/mL, *P* = 0.59).

From these observations, we suggest that hAECs require the presence of Tregs to affect their injury preventative effects following bleomycin administration. Given the previous findings that hAECs reduced macrophage infiltration to the lungs and its association with mitigation of acute injury, we next asked whether hAECs were able to modify macrophage responses to bleomycin-induced injury in *Rag1*^*-/-*^ mice in the absence of mature lymphocytes*.* We determined that hAEC administration alone failed to reduce macrophage infiltration in *Rag 1*^*-/-*^ mice 7 days following bleomycin challenge (Figure 2D, 5.41 ± 1.84 versus 3.76 ± 1.14%, *P* = 0.22). However, the recruitment of macrophages to the lungs was completely abolished in the animals that received hAECs after adoptive transfer of Tregs (0.34 ± 0.12%, *P* = 0.008 versus bleomycin controls). This was not achieved by adoptive transfer of Tregs alone (7.01 ± 1.76%, *P* = 0.07 versus bleomycin controls) or non-Tregs + hAECs (1.72 ± 0.47%, *P* = 0.11 versus bleomycin controls).

### Human amnion epithelial cells promote FoxP3 transcription *in vivo*

**Figure 3:**
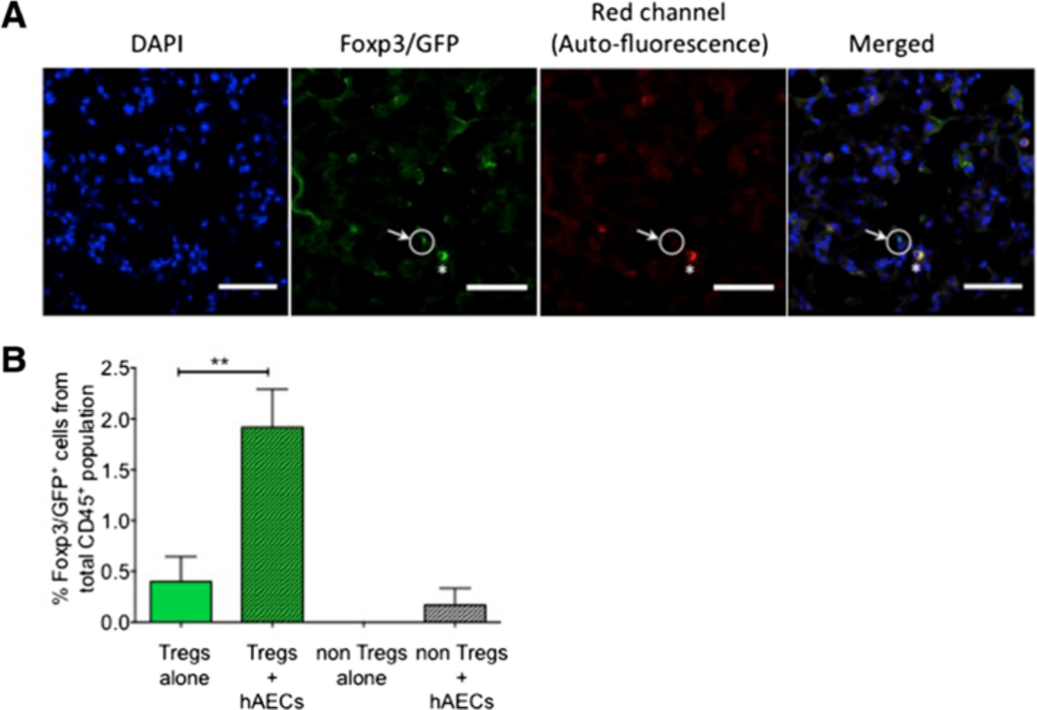
**Human amnion epithelial cells (hAECs) induce transcription of FoxP3** ^**+**^ **in non-regulatory T cells (non-Tregs) and promote Treg expansion** ***in vivo*** **. (A)** Representative images of green fluorescent protein-positive (GFP^+^) cells within the lung parenchyma of *Rag1* ^*-/-*^ mice following adoptive transfer of Tregs (scale bar = 50 μm) (DAPI, blue; FoxP3, green; auto-fluorescence, red). Green staining in the absence of co-localisation with red indicates that the cells are GFP^+^ Tregs rather than autofluorescent cells. **(B)** Co-administration of hAECs and adoptive transfer of Tregs resulted in expansion of Tregs *in vivo* as well as some conversion of non-Tregs into GFP-expressing FoxP3^+^ Tregs (**P <0.01). DAPI, 4′,6-diamidino-2-phenylindole.

In light of the protective effects of hAECs in *Rag1*^*-/-*^ animals that had received non-Tregs (Figure 2A) and the central role that Tregs are thought to have in lung repair [25, 26], we wondered whether hAECs were able to differentiate CD45^+^FoxP3^*-*^ non-Tregs into FoxP3^+^ Tregs. To explore this, we analysed lung sections of *Rag1*^*-/-*^ mice that received non-Tregs with or without hAECs. We detected GFP^+^ cells only in those animals receiving both non-Tregs and hAECs and not in animals receiving non-Tregs alone (representative images in Figure 3A). The overlap of GFP expression with DAPI, concomitant with absent auto-fluorescence in the red channel, confirmed true expression of Foxp3.

We observed a small increase in GFP^+^ cells in the lungs of mice that received non-Tregs (0.17 ± 0.17 versus 0.00 ± 0.00%, *P* = 0.19, Figure 3B). This suggests that a proportion of the non-Tregs may have acquired a FoxP3^+^ phenotype *in vivo* following hAEC administration. Additionally, the percentage of Tregs in the lung was fivefold higher in Treg + hAEC animals compared with Tregs alone (Figure 3B, 1.92 ± 0.37 versus 0.40 ± 0.24%, *P* = 0.0052). These findings are consistent with the administration of hAECs promoting the *in vivo* expansion or recruitment (or both) of Tregs to the lung and the differentiation of non-Tregs into Tregs.

### Human amnion epithelial cells require regulatory T cells to polarise macrophages toward an M2 phenotype

**Figure 4:**
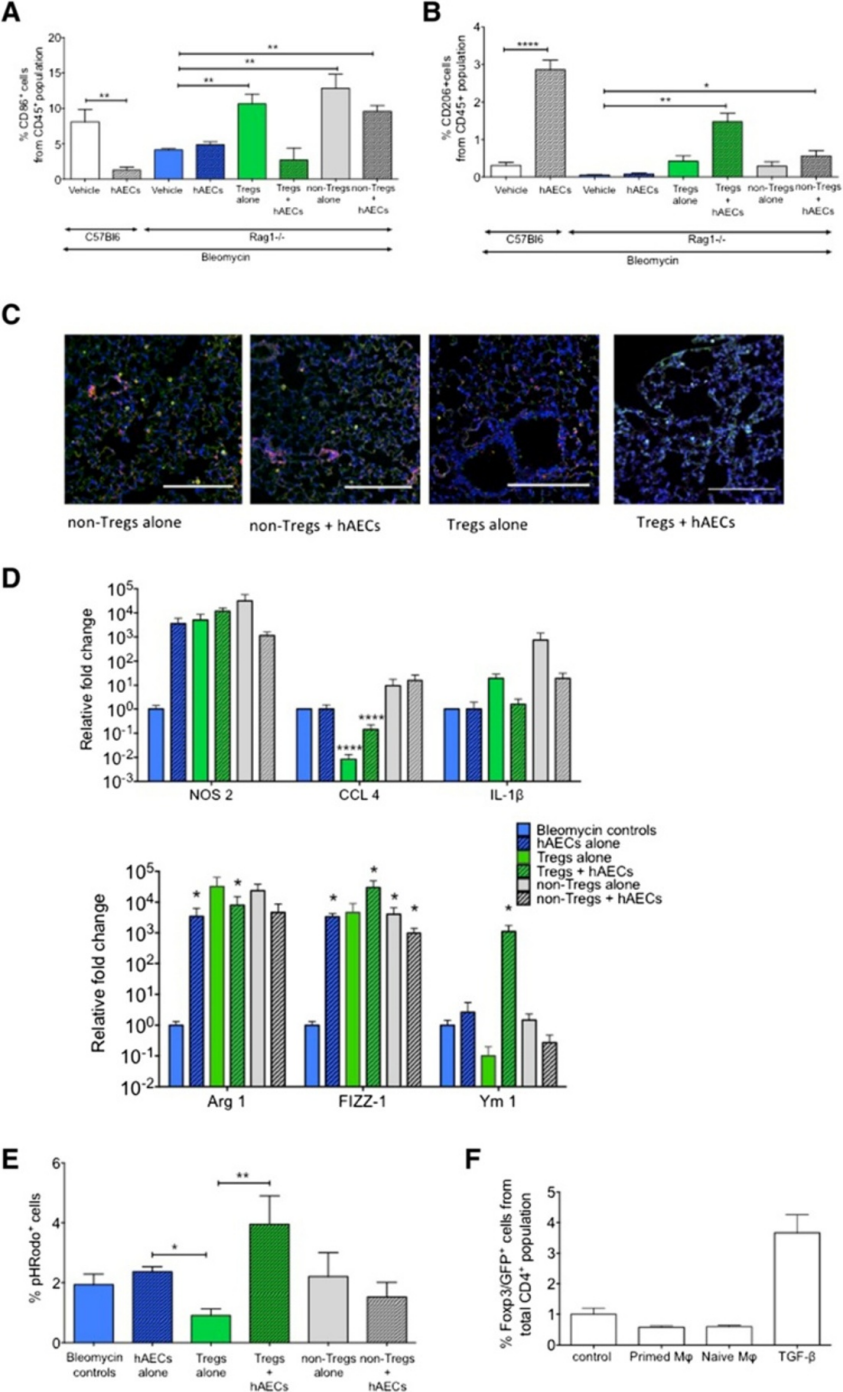
**Human amnion epithelial cell (hAEC) and regulatory T cell (Treg) administration polarise macrophages toward and M2 state in** ***Rag1*** ^***-/-***^ **. (A)** hAEC administration reduced CD86^+^ M1 macrophages in C57Bl6 mice but not in *Rag 1* ^*-/-*^ *mice.* M1 macrophages were significantly increased in the lungs of animals that received Tregs alone, non-Tregs alone, and non-Tregs + hAECs (**P <0.01), but this was not observed in animals that received Tregs + hAECs. **(B)** CD206^+^ M2 macrophages were significantly increased in *Rag 1* ^*-/-*^ animals that received Tregs + hAECs (**P <0.01) and, to a lesser extent, non-Tregs + hAECs (*P <0.05). This trend was similar to that seen in C57Bl6 mice, in which hAEC treatment significantly increased CD206^+^ macrophages in the lungs (****P <0.0001). **(C)** Representative image of CD86^+^ and CD206^+^ macrophages between groups with adoptively transferred cells (scale bar = 200 μm, DAPI, blue; F4/80, green; CD86, pink; CD206, white). These findings were supported by similar changes to the M1- and M2-specific gene expression. **(D)** Co-administration of Tregs and hAECs significantly increased transcription of M2-specific genes found in inflammatory zone protein-1 (*FIZZ-1*) and *Ym-1* (*P* <0.05) (*p<0.05, ****p<0.0001). **(E)** Adoptive transfer of Tregs to *Rag1* ^*-/-*^ mice significantly reduced phagocytic activity of lung macrophages (*P <0.05); however, increased phagocytic activity was observed in macrophages from Treg + hAEC animals compared with Tregs alone (**P <0.001). hAEC priming of macrophages did not alter FoxP3 transcription when the macrophages were co-cultured with CD4^+^ cells. **(F)** CD4^+^ cells were cultured with transforming growth factor-beta (TGFβ) (5 ng/mL) as a positive control. DAPI, 4′,6-diamidino-2-phenylindole.

It is known that hAECs can directly polarise pro-inflammatory M1 macrophages into pro-reparative M2 macrophages *in vitro* without the presence of T cells [10]. Accordingly, we evaluated the polarity of macrophages in the lungs in each of our experimental cohorts. In C57Bl6 mice, hAEC treatment significantly reduced pro-inflammatory M1 (CD86^+^) macrophages (Figure 4A, 1.31 ± 0.42 versus 8.11 ± 1.77%, *P* = 0.004). In *Rag1*^*-/-*^ mice, the percentage of M1 macrophages (CD86^+^) in the lungs was reduced only in animals that received Tregs and hAECs (2.70 ± 1.71 versus 4.17 ± 0.17%, *P* = 0.28). In comparison, we observed increases in the percentage of CD86^+^ M1 macrophages following adoptive transfer of Tregs alone (10.66 ± 1.40%, *P* = 0.008), non-Tregs alone (12.86 ± 1.97, *P* = 0.009), and non-Tregs with hAECs (9.62 ± 0.81%, *P* = 0.001).

In C57Bl6 mice, we observed an increase in M2 macrophages (CD206^+^) following hAEC treatment (Figure 4B, 2.86 ± 0.26 versus 0.31 ± 0.008%, *P* <0.0001). In *Rag 1*^*-/-*^ mice, the percentage of M2 lung macrophages was highest in Treg + hAEC animals where the M2 macrophages were approximately 30-fold higher compared with bleomycin controls (Figure 4B, 1.476 ± 0.23 versus 0.05 ± 0.01, *P* = 0.002). The percentage of M2 macrophages was also significantly increased in non-Treg + hAEC animals compared with bleomycin controls (0.56 ± 0.15%, *P* = 0.02). Representative images of immunofluorescence staining for CD86^+^ (pink) and CD206^+^ (white) macrophages are shown in Figure 4C.

We confirmed the M1/M2 phenotypes of flow-sorted macrophages by quantitative reverse transcription-polymerase chain reaction and phagocytic assay. M1-associated gene, CCL4, was significantly reduced in Treg alone and Treg + hAEC animals (Figure 4D, *P* <0.0001), but NOS-2 and IL-1β were not significantly altered. In contrast, M2-associated genes arginase-1 (*ARG1*), found in inflammatory zone protein-1 (*FIZZ1*), and *YM1* were consistently elevated in lung macrophages flow-sorted from Treg + hAEC animals (Figure 4D, *P* = 0.018, *P* = 0.018, *P* = 0.017, respectively). Furthermore, the highest phagocytic activity was observed in macrophages isolated from Treg + hAEC animals (Figure 4E). Our observations indicate that Tregs play a role in the *in vivo* modulation of macrophages by hAECs.

To explore whether, reciprocally, macrophages had a role in the effects of hAECs on T cells, we co-cultured hAEC-primed macrophages (that is, macrophages polarised by hAECs to an M2 phenotype) with non-Tregs cells. We observed no increase in FoxP3 transcription (Figure 4F), suggesting that *in vivo* Treg induction was unlikely to be mediated by hAEC-primed macrophages.

### Human amnion epithelial cells promote regulatory T cell induction via transforming growth factor-beta

**Figure 5:**
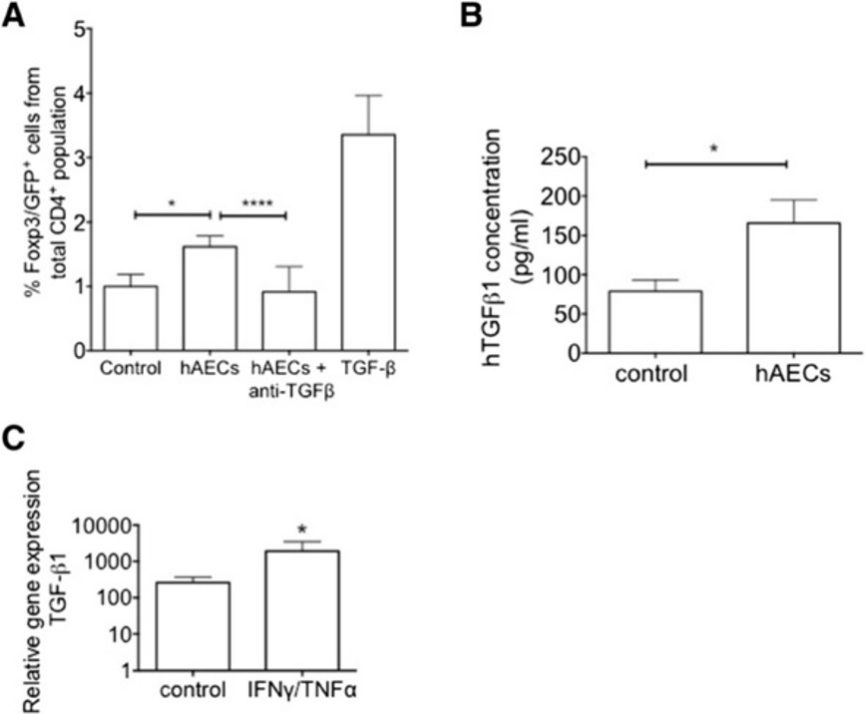
**Human amnion epithelial cells (hAECs) induce regulatory T cell (Treg) maturation via transforming growth factor-beta 1 (TGF-β1). (A)** Co-culture of FoxP3^-^ non-Tregs with hAECs induced FoxP3 transcription and green fluorescent protein (GFP) expression. This was wholly ameliorated by the addition of an anti-TGFβ blocking antibody (1 mg/mL). TGFβ was used as a positive control to induce FoxP3^+^ transcription (*P <0.05, ****P <0.001). **(B)** Levels of human TGF-β1 levels in the supernatant of hAECs were significantly elevated after 5 days co-cultured with non-Tregs (*P <0.05). **(C)** Stimulation with interferon-gamma (IFNγ) and tumour necrosis factor-alpha (TNFα) significantly increased gene expression of *TGF-β1* (*P <0.05).

To determine whether hAECs directly induced FoxP3^+^ transcription in naïve CD4^+^ T cells, we co-cultured hAECs with naïve CD4^+^ T cells isolated from unchallenged healthy mice. FoxP3^+^ transcription was significantly increased after 5 days of co-culture (Figure 5A, 1.0 ± 0.20 versus 1.62 ± 0.17, *P* = 0.02). This was completely prevented by blocking TGFβ signaling (0.32 ± 0.06, *P* <0.0001). hAECs increased secretion of TGFβ1 upon co-culture with CD4^+^ T cells (Figure 5B, *P* = 0.03). This was concurrent with increased TGFβ transcription upon stimulation with inflammatory cytokines interferon-gamma (IFNγ) and tumour necrosis factor-alpha (TNFα) (Figure 5C, *P* = 0.031). These observations are consistent with hAECs increasing TGFβ production when exposed to inflammatory stimuli, which induces Tregs. Given that the release of IL-4 by hAECs has been associated with reduction in monocyte release of pro-inflammatory cytokines [27], we also measured IL-4 released by hAECs following stimulation with IFNγ and TNFα. However, we did not observe any changes to gene transcription or secretion of IL-4 by hAECs (data not shown).

## Discussion

Regenerative medicine—in particular, cell-based therapies—is gaining momentum at a remarkable pace. Over 27,700 clinical trials involving cell therapy were registered on the ClinicalTrials.gov database when this article was under preparation. Clearly, as the potential of cell therapy becomes realised and as research translates from discovery to clinical testing, there is an increasing need to clearly define the mechanisms of stem cell action. In this study, we used a well-established mouse model of lung injury to assess the effect of hAECs on key cellular events that are central to the resolution of inflammation and fibrosis.

We showed that the administration of hAECs increased Treg expansion in the lungs of C57Bl6 mice and this event was coincident with reduced lung injury. Using *Rag1*^*-/-*^ mice, we determined that the presence of Tregs is central to the reparative effects of hAECs *in vivo*. The increase in pulmonary Tregs may be partly attributed to induction of FoxP3 transcription in naïve T cells by hAECs, which we showed to be directed mainly by hAEC-derived TGF-β. Consistent with the limited lung repair observed in *Rag1*^*-/-*^ mice, hAECs were also unable to polarise macrophages from M1 to M2 in the absence of adoptively transferred Tregs. Since macrophage polarisation is a key event in hAEC-mediated lung repair [10, 20], the findings from the present study suggest that Treg expansion/induction is a crucial requirement for hAEC-mediated macrophage polarisation*.* Collectively, these data indicate that Treg induction/expansion is central to hAEC-mediated lung repair, serving as a major driver for macrophage polarisation.

hAECs induced the expansion of endogenous Tregs in *FoxP3-GFP* C57Bl6 mice as well as adoptively transferred Tregs in *Rag1*^*-/-*^ mice. A small proportion of the adoptively transferred non-Tregs differentiated into Tregs *in vivo* when hAECs were co-administered*.* The likely mechanism for this is via hAEC-derived TGFβ. That transcription of TGFβ was increased following exposure to IFNγ and TNFα is worth noting since TGFβ has been shown to be a potent inducer of FoxP3 transcription in naïve CD4^+^ T cells [28]. Another consideration is the process of trogocytosis, where cell-to-cell transfer of HLA-G from hAECs to effector T cells may account for the acquisition of a Treg phenotype independent of FoxP3 transcription [29]. These events may explain the minor improvements in tissue-to-airspace ratio and increase in M2 polarisation in the non-Treg + hAEC group. That adoptively transferred non-Tregs did not wholly restore hAEC-mediated lung repair is likely due to the criteria by which we selected the non-Treg cells. For this study, we selected for CD45^+^FoxP3^-^ cells by flow sorting. One limitation of such a gating strategy is that non-T cells are also included. This includes neutrophils, macrophages, and dendritic cells, which are unable to acquire a FoxP3^+^ phenotype or transdifferentiate into Tregs. The outcomes observed in the present study are likely a reflection of this.

**Figure 6:**
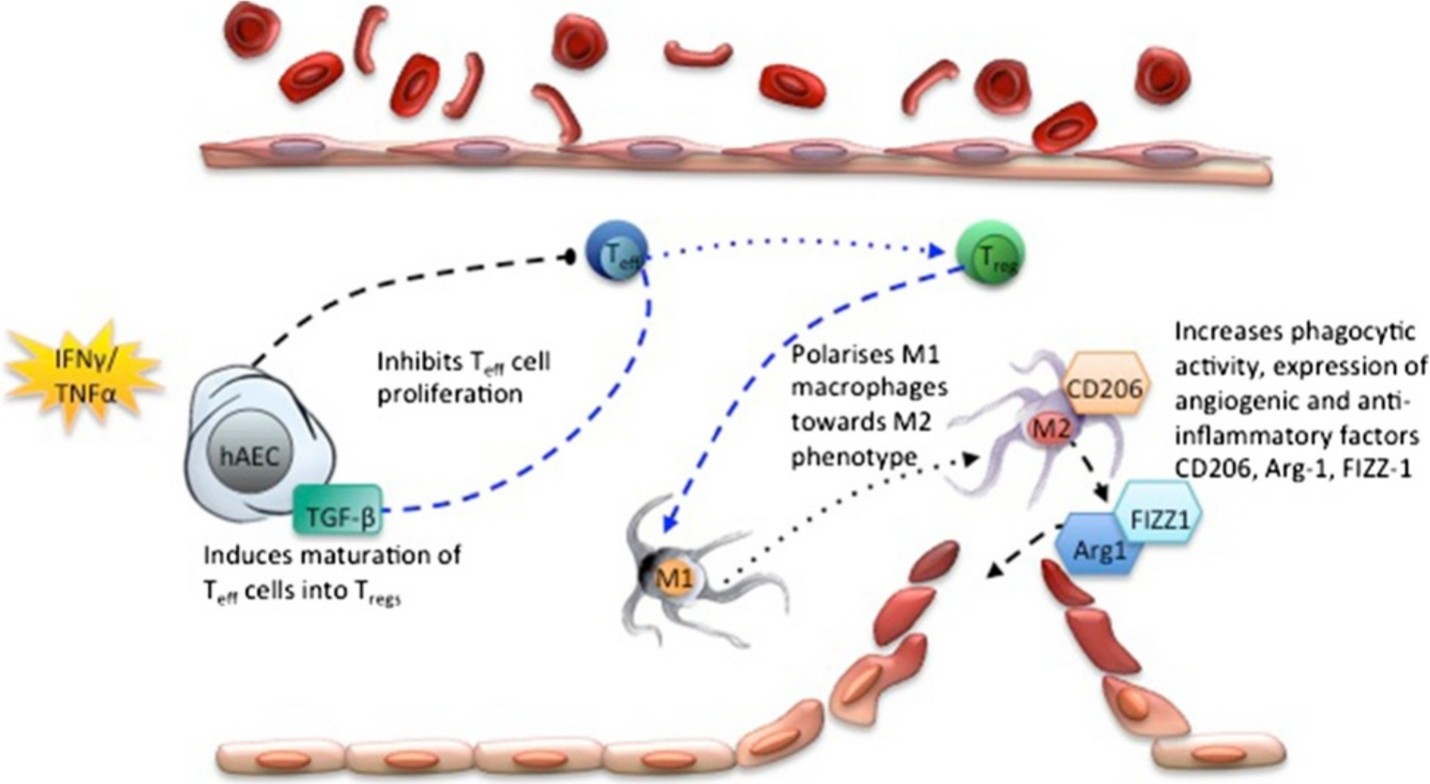
**Schematic illustration of potential mechanisms of human amnion epithelial cell (hAEC)-mediated immune regulation.** Upon stimuli from inflammatory cytokines such as interferon-gamma (IFNγ) and tumour necrosis factor-alpha (TNFα), (i) hAECs synthesize transforming growth factor-beta (TGF-β), thus reducing inflammation by inhibiting effector T-cell proliferation. (ii) TGFβ induces maturation of T cells into regulatory T cells (Tregs), which polarises surrounding resident macrophages toward an M2 phenotype. (iii) M2 macrophages begin the cascade of resolution by increasing their phagocytic activity through mannose receptor, CD206. (iv) Repair is followed closely by pro-angiogenic factors Arg-1 and found in inflammatory zone protein-1 (FIZZ-1), which are also expressed by M2 macrophages.

The reduction in macrophage infiltration and polarisation of macrophages from M1 to M2 was observed only in the cohort of *Rag1*^*-/-*^ animals that received hAECs after adoptive transfer of Tregs (hAEC + Treg). This was surprising because we have previously shown that hAECs can directly influence macrophage polarity and phagocytic activity, at least *in vitro*, without the presence of Tregs [10]. We believe that the different prevailing cytokine environments explain the apparent inconsistency between *in vitro* and *in vivo* observations. *In vivo* in the absence of Treg-released IL-10, the predominant cytokines in the lungs are Th1 cytokines such as TNFα and IL-6 [30] and IFNγ [31]. These promote a Th1 inflammatory environment where M1 macrophages will predominate. The presence of Tregs would then alter the Th 1-Th 2 balance in favour of M2 macrophages. Mature Tregs then release IL-10, which helps to polarise macrophages from M1 to M2 and thus promote lung repair. This suggests that hAEC repair of pulmonary fibrosis may begin with the expansion of the local Treg population, which triggers macrophage polarisation, rather than acting through the direct actions of hAECs. Our proposed model of cellular crosstalk is detailed in Figure 6.

In addition to their effects on macrophage polarisation, Tregs have been reported to play a role in modulating the alveolar inflammatory milieu following lipopolysaccharide (LPS)-induced acute lung injury, in which adoptive transfer of Tregs reduced inflammatory cytokines, including TNFα, monocyte chemoattractant protein-1 (MCP-1), RANTES (regulated on activation, normal T cell expressed and secreted), and IL-6 [32]. In LPS-induced acute lung injury, the adoptive transfer of Tregs resolved fibrosis by reducing fibrocyte recruitment along the CXCL12-CXCR4 axis [25].

With regard to potential cell therapies for lung disease, there are some similarities between hAEC- and MSC-mediated Treg induction. For example, both rely on TGFβ and involve M2 polarised macrophages [33]. However, unlike MSCs, hAECs do not appear to require monocytes as accessory cells [33]. Interestingly, a study by Liu and colleagues [11] using a mouse model of multiple sclerosis showed that the therapeutic effects of hAEC treatment did not correspond to Treg numbers despite increased TGFβ by hAECs. This finding, taken together with the observations in the present study, suggests that the therapeutic mechanisms of hAEC action may be specific to the underlying injury.

The effective clinical translation of bench observations relating to protective or therapeutic benefit of stem cells and stem-like cells will require better understanding of their mechanisms of action. The importance of understanding the mechanism of action of cell-based therapies was highlighted at the NIH-National Heart, Lung, and Blood Institute workshop in November 2012 [23]. Cell-based therapy has been touted as ‘the next pillar of medicine’ [34], given capabilities that extend beyond small molecules and biologics. Understanding the mechanisms by which hAECs exert their reparative or protective effects will aid the development of hAEC-based therapies. Indeed, the elucidation of critical immunological events will aid the design of informative clinical trials.

## Conclusions

hAECs require Tregs for polarisation of macrophage from M1 to M2. In the process of mediating lung repair, hAECs induced Treg expansion and differentiation of T cells to FoxP3^+^ Tregs. Understanding how hAECs, and other stem cells, exert immunomodulatory and reparative effects is important in the clinical translation pipeline.

## Electronic supplementary material

Additional file 1: Figure S1: Gating strategy for flow cytometric sorting of Foxp3^+^ regulatory T cells (Tregs). (A) Cells were gated based on side and forward scatter to exclude cellular debris. (B) Singlets were then selected. (C) CD45^+^ leukocytes were gated from singlets, and FoxP3^+^ Tregs were selected based on green fluorescent protein (GFP) expression. **Figure S2.** Gating strategy for flow cytometric analysis of macrophage polarity. (A) Leukocytes were isolated from the lung of *Rag1* ^*-/-*^ mice and analysed by flow cytometry. Cellular debris was excluded and cells were identified based on side and forward scatter. (B) Doublets were then excluded. (C) This cell population was further gated for total leukocyte population based on CD45 expression. (D) The macrophage population was identified based on double positive expression of pan-macrophage markers; F4/80 and CD11b. (E,F) Gating strategy for polarity of macrophages: Cells that are F4/80^+^CD11b^+^ were gated for macrophage M1- and M2-specific surface markers CD86 and CD206, respectively. Flow cytometric data for M1/M2 macrophages in this manuscript are presented as percentage of CD45^+^ cells. (PDF 1 MB)

## Abbreviations

DAPI: 4′,6-diamidino-2-phenylindole
GFP: green fluorescent protein
hAEC: human amnion epithelial cell
IFNγ: interferon-gamma
IL: interleukin
IN: intranasal
IP: intraperitoneal
IV: intravenous
LPS: lipopolysaccharide
MSC: mesenchymal stromal cell
NIH: National Institutes of Health
OCT: optimal cutting temperature
PGE2: prostaglandin E2
TGFβ: transforming growth factor-beta
TNFα: tumour necrosis factor-alpha
Treg: regulatory T cell.
