# Supplementary figures and images for: Amnion cell-mediated immune modulation following bleomycin challenge: controlling the regulatory T cell response
Source: Stem Cell Res Ther. 2015 Jan 29;6(1):8. doi: 10.1186/scrt542 (PMC4417266; doi:10.1186/scrt542)

Figure S1

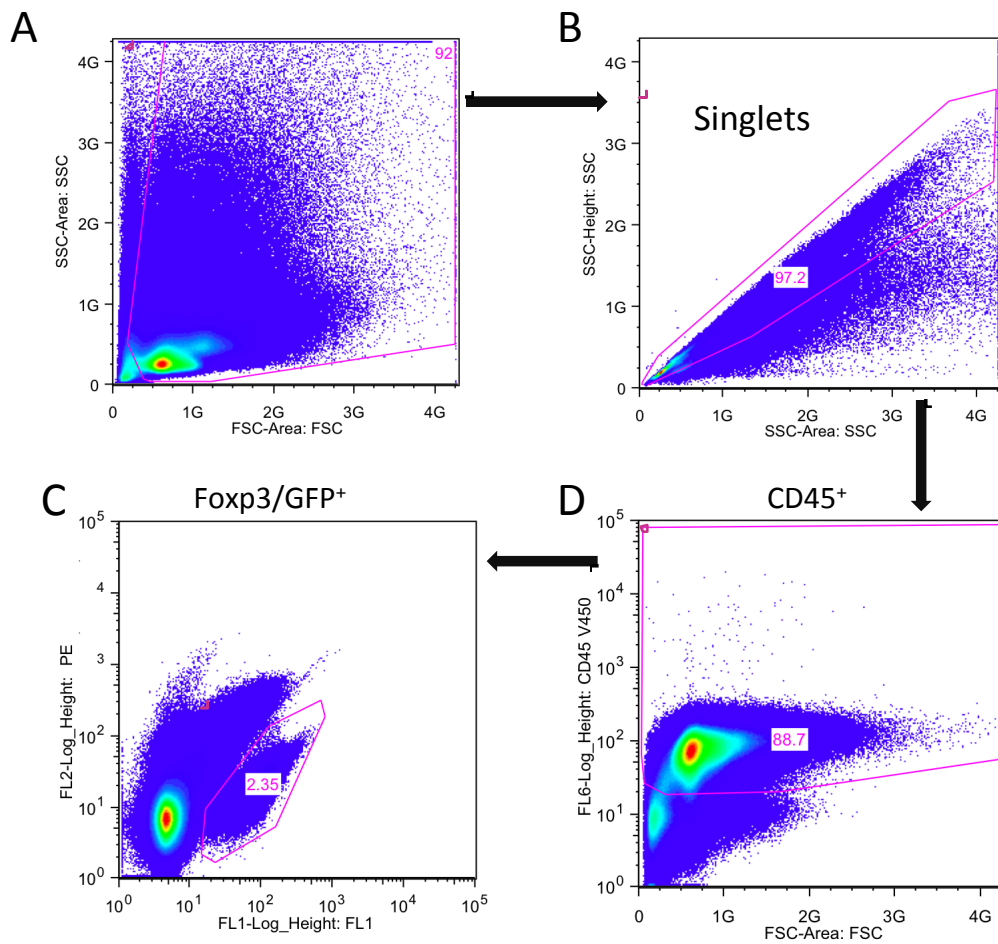

Figure S2

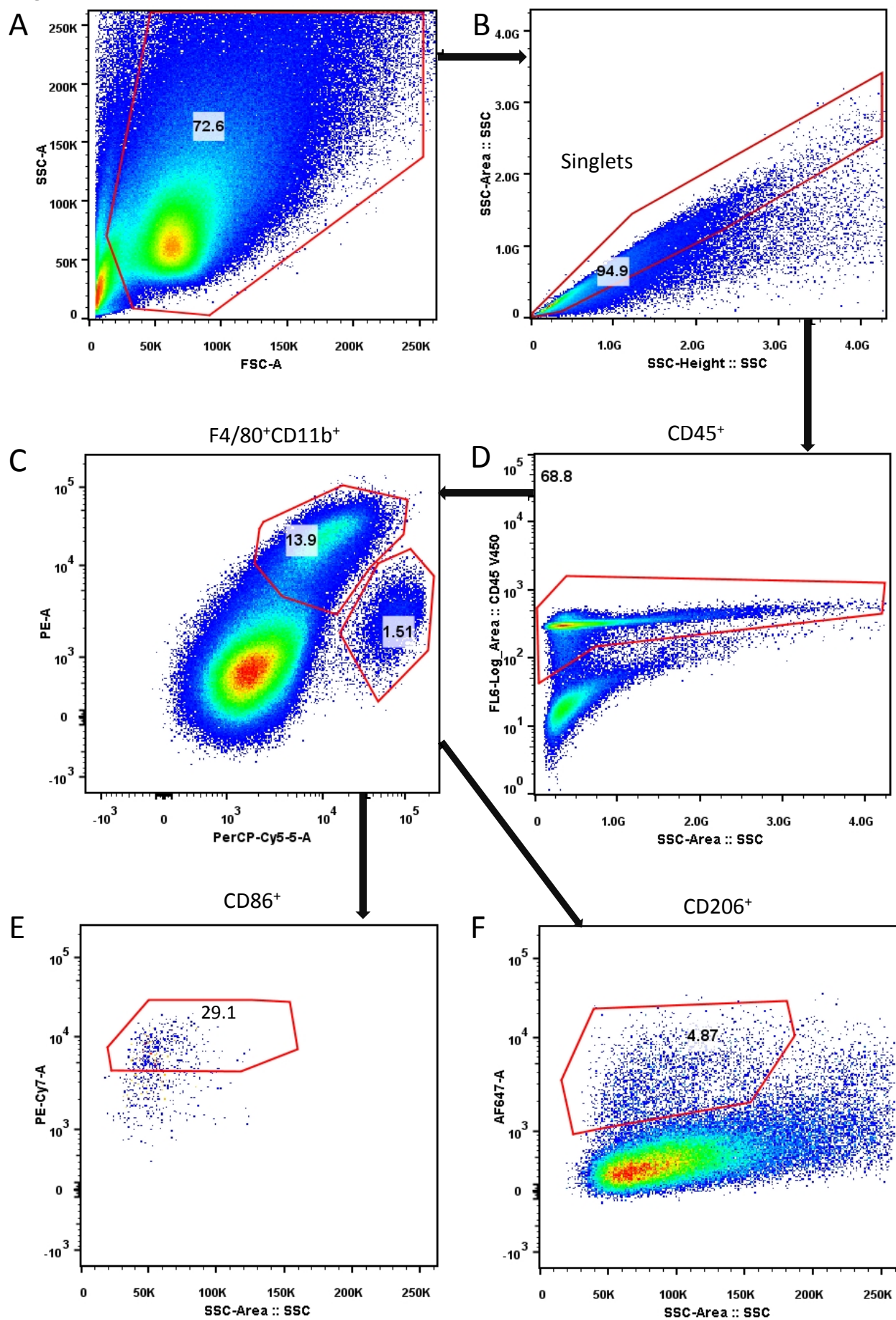

Supplement: Supplementary file 1 — Additional file 1: Figure S1: Gating strategy for flow cytometric sorting of Foxp3+ regulatory T cells (Tregs). (A) Cells were gated based on side and forward scatter to exclude cellular debris. (B) Singlets were then selected. (C) CD45+ leukocytes were gated from singlets, and FoxP3+ Tregs were selected based on green fluorescent protein (GFP) expression. Figure S2. Gating strategy for flow cytometric analysis of macrophage polarity. (A) Leukocytes were isolated from the lung of Rag1 -/- mice and analysed by flow cytometry. Cellular debris was excluded and cells were identified based on side and forward scatter. (B) Doublets were then excluded. (C) This cell population was further gated for total leukocyte population based on CD45 expression. (D) The macrophage population was identified based on double positive expression of pan-macrophage markers; F4/80 and CD11b. (E,F) Gating strategy for polarity of macrophages: Cells that are F4/80+CD11b+ were gated for macrophage M1- and M2-specific surface markers CD86 and CD206, respectively. Flow cytometric data for M1/M2 macrophages in this manuscript are presented as percentage of CD45+ cells. (PDF 1 MB) [file 13287_2014_424_MOESM1_ESM.pdf]
